# Radiation-induced meningiomas in multiple regions, showing rapid recurrence and a high MIB 1 labeling index: a case report and review of the literature

**DOI:** 10.1186/1477-7819-12-123

**Published:** 2014-04-26

**Authors:** Yoshiaki Goto, So Yamada, Shoko M Yamada, Hiroshi Nakaguchi, Katsumi Hoya, Mineko Murakami, Kazuto Yamazaki, Yasuo Ishida, Akira Matsuno

**Affiliations:** 1 Department of Neurosurgery, Teikyo University Chiba Medical Center, 3426-3 Anesaki, Ichihara-City, Chiba 299-0111, Japan

**Keywords:** Radiation-induced meningioma, cranial irradiation, childhood leukemia, multiple lesions, MIB-1 labeling index

## Abstract

Combined chemotherapy and prophylactic cranial irradiation has improved the prognosis of children with acute leukemia. However cranial irradiation carries a latent risk of the induction of secondary intracranial tumors. We encountered a patient who developed multiple intracranial radiation-induced meningiomas (RIMs) 25 years after prophylactic cranial irradiation for the treatment of acute leukemia in childhood. The patient had 3 intracranial lesions, 1 of which showed rapid growth within 6 months; another of the tumors also enlarged within a short period. All of the tumors were surgically treated, and immunohistochemistry indicated a high MIB-1 labeling index in each of the multiple lesions. In the literature, the MIB-1 labeling indices of 27 tumors from 21 patients were examined. Among them, 12 recurrent tumors showed higher MIB-1 labeling indices compared to the MIB-1 labeling indices of the non-recurrent tumors. Overall, 11 of the patients with RIM had multiple lesions and 8 cases developed recurrence (72.7%). RIM cases with multiple lesions had higher MIB-1 labeling indices compared to the MIB-1 labeling indices of cases with single lesions. Collectively, these data showed that the MIB-1 labeling index is as important for predicting RIM recurrences, as it is for predicting sporadic meningioma (SM) recurrences. RIMs should be treated more aggressively than SMs because of their greater malignant potential.

## Background

Children with acute leukemia have been treated with the combination of chemotherapy and prophylactic cranial irradiation. This combination therapy was found to improve their prognoses dramatically [1]. However, long after the irradiation exposure, some survivors developed secondary intracranial tumors such as glioma, sarcoma, and meningioma [1,2]. In this paper, we report a patient who developed multiple intracranial meningiomas, all of which had aggressive clinical features and high MIB-1 labeling indices, 25 years after prophylactic cranial irradiation to treat acute leukemia in childhood. Radiation-induced meningiomas (RIMs) with multiple lesions, all of which have high MIB-1 labeling indices, have not been reported yet. The correlation between the clinical features of RIMs and the MIB-1 labeling index are discussed, along with a review of the literature.

## Case presentation

**Figure 1:**
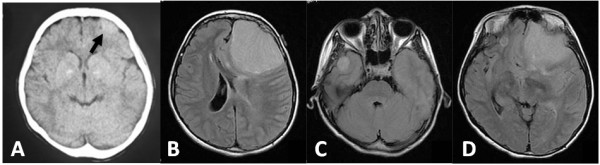
**Preoperative axial computed tomography (CT) and fluid-attenuated inversion recovery (FLAIR) magnetic resonance imaging (MRI). (A)** CT image showing a tumor approximately 1.5 cm in diameter in the left frontal convexity (arrow). (**B)** Tumor in the left frontal convexity. The tumor mass and perifocal edema caused a shift of the brain to the right side. (**C)** Tumor in the right middle cranial fossa. (**D)** Right frontal base tumor (cranial fossa) and the tumor in the left frontal convexity.

**Figure 2:**
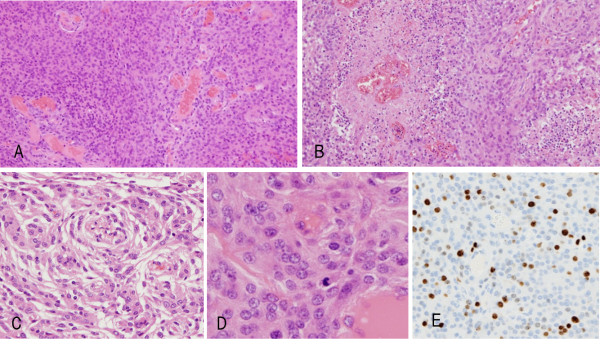
**Histological findings.** The left-frontal meningioma tissue shows hypercellular lesions **(A)**, with focal necrosis **(B)** (original magnification, ×100), and a whorl formation **(C**) (original magnification, ×100). Frequent mitoses **(D)** (original magnification, ×200) are also evident. The immunohistochemical profile revealed approximately 20% Ki-67-positivity **(E)** (original magnification, ×100).

A 28-year-old woman presented with a 1-week history of headache, nausea, and vomiting. The patient had a medical record of acute lymphoblastic leukemia at 3 years of age. She had received a chemotherapy regimen that comprised vincristine, methotrexate, L-asparaginase, cytosine arabinoside, and 6-mercaptopurine, as well as whole-brain irradiation at a total dose of 18 Gy. The combined treatment brought the patient into clinical remission, and the patient was followed up until she was 18 years old with no relapse of the disease. Thereafter, she was lost from oncologic follow up. Six months before her visit to our hospital, she consulted another doctor about a mild head injury. Computed tomography (CT) of the brain showed no traumatic findings but incidentally revealed a small lesion in the left frontal convexity (Figure 1). Six months later, she suffered from headache, nausea, and vomiting. A further CT examination revealed rapid enlargement of the lesion, and she was referred to our institution. Magnetic resonance imaging (MRI) of the brain revealed two other small-mass lesions in the right middle and frontal fossae (Figure 2). Surgical resection of the left frontal-mass lesion was performed. During surgery, the tumor was found to adhere to the dura mater of the left frontal convexity and the wall of the superior sagittal sinus. The tumor was extensively removed, and the dural attachment was cauterized (Simpson grade 2). Histopathological examination revealed frequent mitoses, hypercellularity, and focal necrosis in the tumor specimens. Immunohistochemical analysis revealed an MIB-1 labeling index of approximately 20%. The pathological diagnosis was atypical meningioma.

Postoperatively, the patient presented with neither neurological deficits nor radiological evidence of recurrence at the surgical site. Six months after surgery, a follow-up brain MRI revealed enlargement of the right middle fossa lesion. Thus, the two lesions on the right side were resected. Histopathological examination identified both tumors as meningothelial meningioma with MIB-1 labeling indices of approximately 10% and 15%, respectively. The postoperative clinical course was uneventful, and the patient developed no neurological deficits. Nine months after the first surgery, a follow-up MRI revealed a tumor recurrence in the left parasagittal region. Twelve months after the first surgery, a follow-up MRI identified a recurrence of the left frontal convexity meningioma in the frontal superior sagittal sinus and the frontal convexity dura matter. During the third operation, the tumor was found to have invaded into the frontal superior sagittal sinus, where it had attached to the bridging vein. The tumor was resected extensively and the dural attachment was cauterized. After surgery, the patient developed no neurological deficits.

## Discussion

Since Mann *et al*. reported the first case of RIM in 1953 [3], more than 750 cases have been reported in the English-language literature. Several features that distinguish between RIM and sporadic meningioma (SM) have been indicated. SM is known as a benign tumor that accounts for approximately 20% of all intracranial tumors. SM tends to occur during middle age and has a female predominance [4-6]. Benign meningioma accounts for 90% of SM cases. Atypical meningioma and anaplastic meningioma each account for 5% and 3% of SM cases, respectively [7]. Roser *et al*. analyzed 600 cases of SMs and revealed that 91% of SMs are classified as World Health Organization (WHO) grade 1, with a mean MIB-1 labeling index of 3.28%. Additionally, 7% and 2% of SM cases are classified as WHO grade 2 (mean MIB-1 labeling index, 9.95%) and grade 3 (mean MIB-1 labeling index, 12.18%), respectively [8].

RIM tends to occur in patients who are younger at the time of clinical presentation [9] and has an increased incidence of multiplicity. Strojan *et al*. reported 126 secondary meningiomas, 10 (8%) of which had multiple lesions [10]. Another report estimated that nearly 16% of RIM cases have multiple mass lesions [11] and a higher rate of atypical or aggressive forms. Al-Mefty *et al*. reported that approximately 31% of RIM cases were atypical meningiomas with a higher recurrence rate [12], which was also suggested by several other investigators [13,14]. A pathological study of RIMs revealed that RIMs presented with more malignant features than SMs, namely, hypercellularity, pleomorphism, increased mitotic figures, and necrotic changes [15,16].

Immunohistochemical analysis is also useful for evaluating the malignant potential and recurrence rate of SMs [17]. The proliferative marker, MIB-1 labeling index, is a useful method for this purpose. However, several reports state that the MIB-1 labeling index is not an appropriate method with which to evaluate the malignant potential and recurrence rate of RIMs [12,18]. All of the lesions in our RIM case had high MIB-1 labeling indices, and the tumor recurred postoperatively within 1 year. Thus, we reviewed the literature that evaluated the MIB-1 labeling index for RIMs.

From the literature, the MIB-1 labeling indices of 27 tumors from 21 patients were examined (Table 1) [10,12,18-20]. Among these, 12 recurrent tumors had higher MIB-1 labeling indices than the MIB-1 labeling indices of the non-recurrent tumors (mean ± SD); recurrent tumors versus non-recurrent tumors, 9.1 ± 5.1 versus 4.9 ± 3.9, *P* <0.05). Eighteen tumors were classified as WHO grade 1, with a mean MIB-1 labeling index of 4.66%, and four tumors were grade 2 (17.4%) with a mean MIB-1 labeling index of 14.25%. Among the 21 RIM cases, 11 had multiple lesions and 8 cases developed recurrence (72.7%). RIM cases with multiple lesions had higher MIB-1 labeling indices than the MIB-1 labeling indices of those with single lesions (mean ± SD; multiple lesion tumors versus single lesion tumors, 8.6 ± 5.2 versus 3.6 ± 1.8, *P* <0.01). Collectively, these data show that the MIB-1 labeling index is as important for predicting the recurrence of RIMs, as it is for predicting the recurrence of SM. Noticeably, the pathological features of RIMs with multiple lesions are not always consistent. The growth rate of each lesion varies [15,16,19], and some lesions are managed conservatively [2,10,11]. RIM should be treated more aggressively than SM because of its greater malignant potential. Undoubtedly, surgical resection is the first choice of treatment. For RIM located in surgically inaccessible regions, including the skull base or around the brain stem, radiosurgery, including gamma knife surgery, is another treatment option [21,22].

**Table 1:** Summary of reported cases of radiation-induced meningioma

**Case**	**Age (years)/sex**	**Histology**	**Cranial irradiation TD**	**Age (years), latency**	**WHO grade/histology**	**Recurrence**	**Multiple**	**Ki-67 (%)**	**Author name, year**
1	1/M	Cerebellar medulloblastoma	30 Gy to WB, 10.5 Gy to tumor	12, 11	1	CR 12 years	No	1.8	S.Nishio 1998
2	2/M	ALL	24 Gy to WB	16, 14.5	1	CR 3 years	No	3.7	S.Nishio 1998
3	23/F	Astrocytoma	60 Gy to humor	37, 14	1	Progression of the primary disease, dead after 22 months	No	1.7	S.Nishio 1998
4	17/F	Ependymoma	30 Gy to WB and 30 Gy to tumor	38, 21	1	CR 3 years	No	6.9	S.Nishio 1998
5	10/F	Medulloblastoma	30 Gy to WB and 29.5 Gy to tumor	41.5, 31.5	Benign, transitional	No	No	1	P.Strojan 2000
6	11/M	Medulloblastoma	31.5 Gy to WB and 24 Gy to tumor	35, 24	Benign, meningotheliomatous	No	Yes (3)	9	P.Strojan 2000
					Benign, transitional			3	
					?			?	
7	9/F	ALL	10 Gy to WB	29, 20.5	Benign, transitional	Yes (3)	Yes (3)	2	P.Strojan 2000
	13.5	ALL, relapse	24 Gy to WB	30, 21.5	Atypical, transitional			7	
				31, 22.5	Atypical, transitional			15	
				31.5, 23	?			?	
8	4/M	ALL	18 Gy to WB	13, 9.5	Atypical, fibrous	Yes (1)	Yes (2)	15	P.Strojan 2000
				14.5, 11	Benign, fibrous			1	
				18.5, 15	?			?	
9	3/M	NHL	19.5 Gy to basal skull	15.5, 12.5	Atypical, fibrous	No	No	3	P.Strojan 2000
	3.5	NHL, relapse	24 Gy to basal skull						
10	13/M	Craniopharyngiom	HD Gy to tumor	44, 31	Atypical	No	Yes (3)	?	Al-Mefty 2004
				47, 34	Benign, meningotheliomatous			1	
				53, 41	?			?	
11	12/M	NHL	HD Gy to WB	34, 22	Benign, meningotheliomatous	Yes (1)	Yes (2)	3	Al-Mefty 2004
				37, 25	?			?	
12	3/M	Medulloblastom	HD (50 Gy)	9, 6	Clear cell	Yes (1)	Yes (NA)	7	Samer KE, 2012
13	7/F	AML	MD (16 Gy)	14, 7	Meningothelial, chordoid	Yes (1)	Yes (NA)	10	Samer KE, 2012
14	5/M	Ependymoma	HD (55)	18, 13	Meningothelial	Yes (1)	No	6	Samer KE, 2012
15	7/M	ALL	MD (14 Gy)	16, 9	Fibroblastic	No	No	4	Samer KE, 2012
16	2/M	Medulloblastoma	HD (50 Gy)	13, 11	Atypical meningotherial, xanthomatou	Yes (1)	Yes (NA)	6.2	Samer KE, 2012
17	7/F	ALL	HD (30 Gy)	17, 10	Meningothelial, chordoid	Yes (2)	Yes (NA)	7.2	Samer KE, 2012
18	4/F	Medulloblastoma	HD (50 Gy)	17, 13	Meningothelial, fibroblastic	No	Yes (2)	6.9	Samer KE, 2012
19	5/M	Ependymoma	HD (55 Gy)	17, 12	Atypical meningothelial, rhabdoid	Yes (1)	Yes (NA)	10.4	Samer KE, 2012
20	2/M	Ependymoma	HD (55 Gy)	10, 8	Xanthomatous	No	No	5	R.Ijiri 2000
21	3/M	ALL	18 Gy to WB	28, 25	Benign, meningotheliomatous	Yes (1)	Yes (3)	10	Current case report
					Benign, meningotheliomatous			15	
					Atypical			20	

WB, whole brain; CR, complete remission; ?, no surgical treatment and unknown pathological features; HD, high dose (greater than 20 Gy); M, male; F, female; ALL, acute lymphoblastic leukemia; NHL, non-hodgkin lymphoma; AML, acute myelogenous lymphoma; TD, total dose; NA, not available.

## Conclusion

We experienced a case of multiple RIMs with rapid tumor progression and recurrence within a short period, and the MIB-1 labeling indices in all tumors exceeded 10%. The MIB-1 labeling index is an important index for evaluating the malignant potential and recurrence of both RIMs and SMs. RIMs have a greater malignant potential than SMs, and thus, a more aggressive treatment is required for RIMs.

## Consent

We explained to the patient and family about the RIMs and written informed consent was obtained from them for publication of this Case report and any accompanying images. A copy of the written consent is available for review by the Editor-in-Chief of this journal.

## Abbreviations

CT: computed tomography; MRI: magnetic resonance imaging; RIM: radiation-induced meningioma; SM: sporadic meningioma; WHO: World Health Organization.

## Competing interests

The authors declare that they have no competing interests.

## Authors’ contributions

YG and SY collected and analyzed the data. YG, SMY, and KH developed the concept and designed the paper. MM and KH supervised and commented on the paper. KY and YI conducted pathological examinations and commented on the paper. All of the authors read and approved this paper. AM gave final approval of this article for submission.

## Authors’ information

YG and SY are staff assistants, YMS, MM, and KH are associate professors, and AM is professor of the Department of Neurosurgery at Teikyo University Chiba Medical Center.
